# Choroidal manifestations of non-ocular sarcoidosis: an enhanced depth imaging OCT study

**DOI:** 10.1186/s12886-024-03463-0

**Published:** 2024-04-26

**Authors:** Ye Eun Han, Jaehyuck Jo, Ho Cheol Kim, Junyeop Lee

**Affiliations:** 1 Department of Ophthalmology, Asan Medical Center, University of Ulsan College of Medicine, 88, Olympic-Ro 43-Gil, Songpa-Gu, Seoul, 05505 Republic of Korea; 2 Department of Pulmonology and Critical Care Medicine, Asan Medical Center, University of Ulsan College of Medicine, Seoul, South Korea; 3 Translational Biomedical Research Group, Asan Institute for Life Science, Asan Medical Center, Seoul, South Korea

**Keywords:** Sarcoidosis, Choroidal thickness, Choroidal vascularity index, Enhanced depth imaging, Spectralis-domain optical coherence tomography

## Abstract

**Background:**

Although choroidal thickening was reported as a sign of active inflammation in ocular sarcoidosis, there has been no research on the choroidal changes in non-ocular sarcoidosis (defined as systemic sarcoidosis without overt clinical signs of ocular involvement). Therefore, this study aimed to investigate choroidal structural changes in patients with non-ocular sarcoidosis.

**Methods:**

This retrospective case–control study was conducted at Asan Medical Center, a tertiary referral center. We evaluated 30 eyes with non-ocular sarcoidosis and their age- and spherical equivalent-matched healthy control eyes. The subfoveal choroidal thickness, area ratio (Sattler layer-choriocapillaris complex [SLCC] area to Haller layer [HL] area), and choroidal vascularity index (CVI, luminal area to choroidal area) were analyzed using enhanced depth imaging in optical coherence tomography. Systemic and ocular factors associated with the choroidal thickness were investigated.

**Results:**

Compared with the healthy control group, the non-ocular sarcoidosis group had significantly thicker subfoveal choroid (total and all sublayers [SLCC and HL]) and lower area ratio. There were no significant differences in the CVIs at all sublayers between groups. In the non-ocular sarcoidosis group, eyes under oral steroid treatment had thinner choroid than eyes under observation. In the control group, eyes with older age and more myopic spherical equivalent had thinner choroidal thickness.

**Conclusion:**

Total and all sublayers of the subfoveal choroid were significantly thicker without significant vascularity changes in non-ocular sarcoidosis eyes than in healthy control eyes. The degree of choroidal thickening was disproportionally greater at HL than at SLCC. These characteristic choroidal changes may be the subclinical manifestations in non-ocular sarcoidosis.

## Background

Sarcoidosis is a chronic multisystem disorder of unknown origin defined by non-caseating granulomas with the accumulation of T-lymphocytes [1]. Although the most commonly affected organs are the lungs and intrathoracic lymph nodes, almost any organ can be affected [2]. Ocular involvement is the second most common extrathoracic manifestation in 25–60% of patients with systemic sarcoidosis [2, 3]. Ocular sarcoidosis can also involve any part of the eye and its adnexal tissue [2, 3]. The most common ocular manifestation is uveitis, followed by conjunctival nodules [2, 3]. Reportedly, approximately 25% of the patients initially diagnosed with pulmonary sarcoidosis were found to have bilateral uveitis [4]. The typical types of uveitis associated with sarcoidosis are granulomatous and bilateral; it can be anterior-, intermediate-, posterior-, and pan-uveitis [5]. Posterior uveitis involving the retina and/ or choroid accounts for 5–28% of ocular sarcoidosis [5].

Previous studies have shown that sarcoidosis-related uveitis results in changes in choroidal thickness in addition to retinal abnormalities using enhanced depth imaging in spectral-domain optical coherence tomography (EDI SD-OCT) [6, 7]. Interestingly, changes in choroidal thickness were also found even in eyes without overt intraocular inflammation in patients with Behçet's disease, another multiorgan-involving inflammatory disease [8]. It suggests systemic inflammatory diseases may have subclinical manifestations in the choroid without apparent clinical signs of intraocular involvement. However, to our knowledge, there has been no research regarding choroidal changes in eyes with systemic sarcoidosis without overt clinical signs of ocular involvement (defined as non-ocular sarcoidosis). This study aimed to investigate choroidal structural changes in non-ocular sarcoidosis using EDI SD-OCT.

## Methods

### Patients

This retrospective observational case–control study was conducted at Asan Medical Center, a tertiary referral center in Seoul, South Korea. We retrospectively reviewed the medical records of all patients referred for ophthalmic examination under the diagnosis of systemic sarcoidosis from March 2020 to July 2021. Among them, eyes without ophthalmological signs for ocular involvement based on the International Workshop on Ocular Sarcoidosis criteria [9] were included. To thoroughly exclude intraocular inflammation in sarcoidosis, which may only be detectable through angiogenic evaluation [4], we additionally assessed wide-field fluorescein angiography [wFA] and indocyanine green angiography [wICGA]. After that, their age- and spherical equivalent (SE)-matched healthy controls were selected among those who visited our clinic for retinal screening. The OCT image acquisition times were also matched within a 2-h difference to minimize the confounding effect of the diurnal variation of choroidal thickness [10]. Eyes with any history of ocular surgery (including refractive or cataract surgery to make SE reflect axial length), incomplete ocular evaluations required for this study, and other ocular or systemic diseases (except sarcoidosis) that possibly cause intraocular complications were excluded. If both eyes were qualified for the study, the right eye of each patient was included in the analysis.

### Ocular and systemic evaluation

The ocular examinations were assessed by retinal specialists (Y.E.H, J. J. and J. L.), which included best-corrected visual acuity (BCVA), intraocular pressure by non-contact tonometry (Topcon CT-60, Topcon; Tokyo, Japan), SE from manifested refraction, slit-lamp biomicroscopy, funduscopic examination, and EDI SD-OCT (Heidelberg Engineering GmbH; Heidelberg, Germany), for both the sarcoidosis and control groups. Additionally, wFA and wICGA (HRA-2; Heidelberg Engineering, Heidelberg, Germany) were assessed for the sarcoidosis group. Diagnosis (based on biopsy and radiological findings) and treatment regimen of systemic sarcoidosis were determined by a pulmonologist (H.C.K.). Blood tests (including Angiotensin-converting enzyme [ACE] and C-reactive protein [CRP]) were conducted on the day of referral to the ophthalmologists. Disease duration was defined as the time gap from the preliminary diagnosis of systemic sarcoidosis to the first day of the ophthalmologic examinations.

### SD-OCT acquisition and analysis

**Fig. 1:**
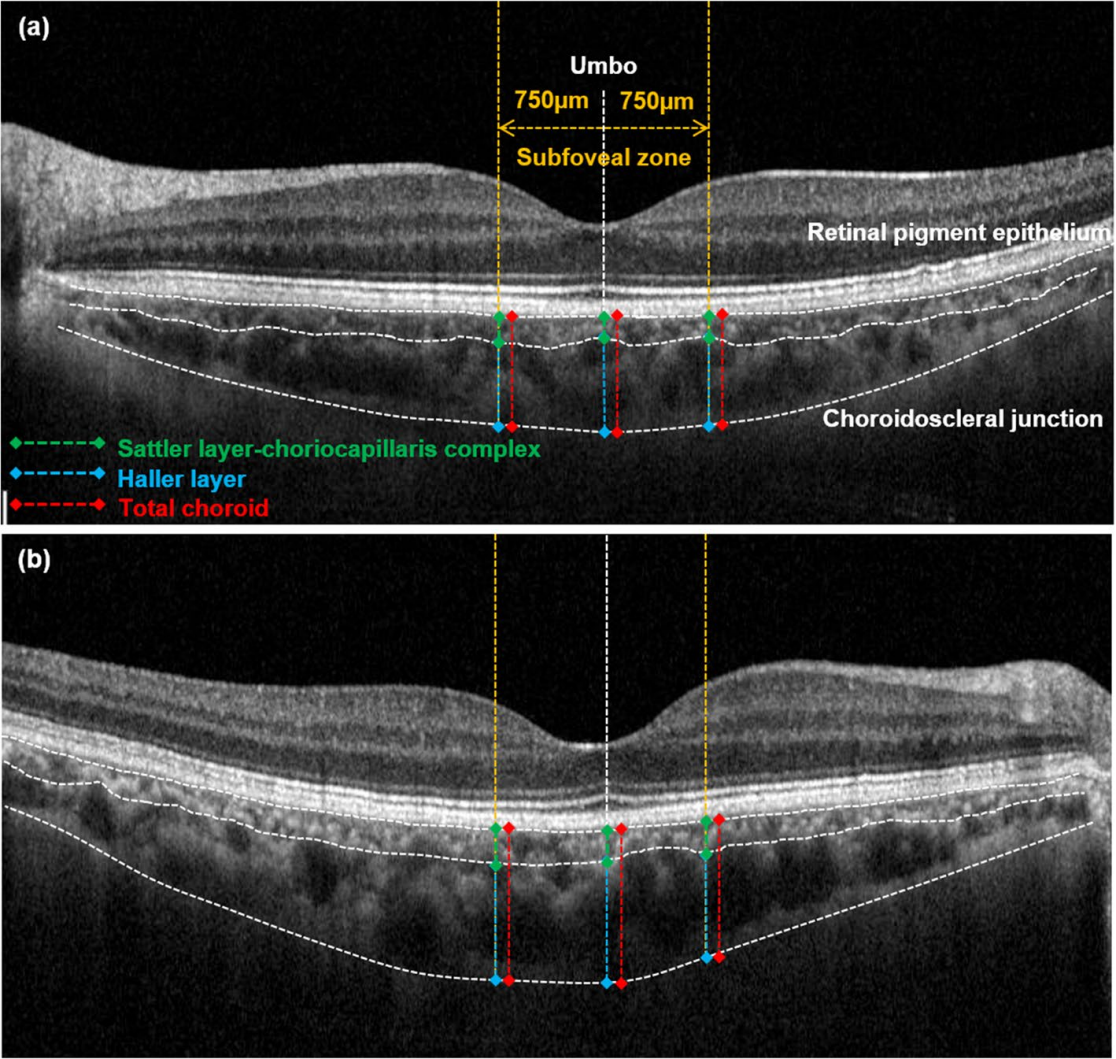
Representative enhanced depth imaging in spectral domain optical coherence tomography of (**a**) healthy control eye and (**b**) non-ocular sarcoidosis eye

SD-OCT with enhanced depth imaging (EDI) protocols [11] obtained detailed and measurable images of the choroid. Among horizontal raster pattern scans (30° × 5°, 9.0 mm × 1.5 mm field), umbo-centered images were selected for the analysis. As shown in Fig. 1, the choroid was defined as the space between the retinal pigment epithelium and choroidoscleral junction. The choroid was subdivided into Sattler layer-choriocapillaris complex (SLCC; < 100-μm sized small-to-medium vascular luminal spaces plus choriocapillaris) and Haller layer (HL; ≥ 100-μm sized large vascular luminal spaces) [12–14]. The subfoveal zone was defined as the 750-μm distance around the umbo. Using built-in software (Heidelberg Eye Explorer version 1.10.1.0; Heidelberg Engineering), the subfoveal choroidal thickness (the mean value of the perpendicular thickness at three points; the umbo and 750-μm intervals [right and left]) of the total and each sublayer was manually measured. Additionally, the area of each sublayer at the subfoveal zone was also measured by manual tracing, and their area ratio was calculated by dividing SLCC area by HL area, indicating SLCC occupying area percentage from HL.

**Fig. 2:**
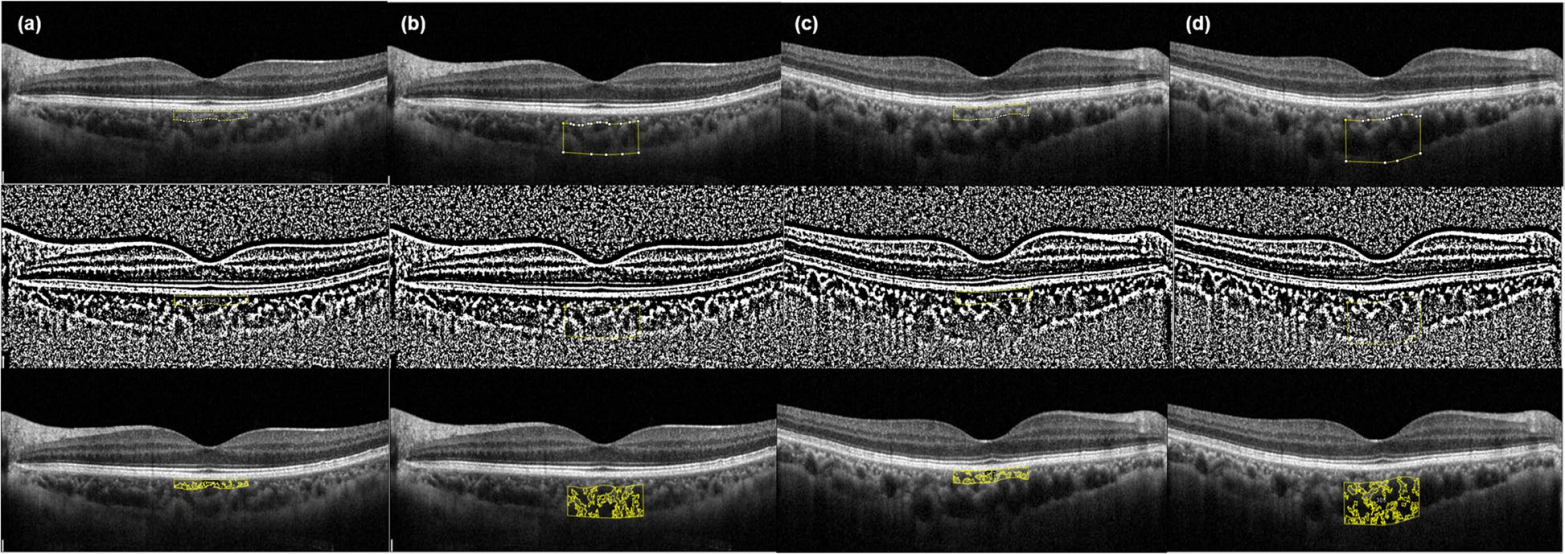
Measurement of choroidal vascularity index in healthy control eye and non-ocular sarcoidosis eye. **a** Sattler layer-choriocapillaris complex and (**b**) Haller layer in healthy control eye and (**c**) Sattler layer-choriocapillaris complex and (**d**) Haller layer in eye with non-ocular sarcoidosis

After importing SD-OCT images into ImageJ software (version 1.52, Wayne Rasband, National Institutes of Health; Bethesda, MD, USA), the choroidal vascularity index (CVI) was calculated to assess the vascularity status of each sublayer of the choroid (Fig. 2). First, the manually subdivided SLCC and HL were binarized using Niblack’s auto-local threshold. After that, the selection of dark pixels (representing the luminal area) by color threshold was performed, and CVI was calculated by dividing the luminal area by the total choroidal area [15, 16]. All manual segmentation and measurements were carried out by two independent examiners (Y.E.H. and J.J.), and averaged data were used in the final analysis. The degree of inter-examiner agreement in each measurement was assessed by Interclass correlation coefficient (ICC) and shown by Bland–Altman plot.

### Statistical analysis

Descriptive statistics were demonstrated in numbers and percentages for categorical variables and means ± standard deviations for continuous variables. A Student’s t-test and Chi-squared test were used to evaluate the demographic and ocular differences between the groups depending on the variable types. Univariate and multivariable linear regression analysis and Chi-squared test were used to estimate the relationship between choroidal thickness and the demographic and ocular factors depending on their variable types. We considered a *p*-value of less than 0.05 as statistically significant and less than 0.10 as borderline significant. All of the statistical analyses were performed using SPSS software (version 21.0, IBM Corp.; Armonk, NY, USA).

## Results

During the study period, we screened 71 systemic sarcoidosis patients, and of these, 19 patients (26.8%) were diagnosed with ocular sarcoidosis. Among 52 non-ocular sarcoidosis patients, 30 patients met the study eligibility criteria. Therefore, a total of 30 eyes of 30 non-ocular sarcoidosis patients and their age- and spherical equivalent-matched healthy controls were included in this study. The demographics and ocular characteristics are shown in Table 1. The mean age of the patients with non-ocular sarcoidosis was 55.13 ± 11.58 years, with a male-to-female ratio of 11:19. The mean logMAR BCVA, SE, and intraocular pressure were 0.03 ± 0.05, -0.71 ± 1.93 diopters and 15.73 ± 2.40 mmHg, respectively. Twenty-seven patients (90%) were diagnosed with pulmonary sarcoidosis; three patients (10%) were extrapulmonary sarcoidosis (skin involvement [*n* = 3, 10%]). During 16.10 ± 26.18 months of systemic sarcoidosis duration, eleven patients (36.66%) have received medical treatments (oral steroid, prednisolone 10-40 mg/day [*n* = 6, 20.00%] and methotrexate 15 mg/day [*n* = 5, 16.66%]), while others (*n* = 19, 63.33%) were under observation. The mean referral-to-ophthalmologic screening duration was 33.38 ± 41.12 days. The healthy control group showed no significant difference in baseline demographic and ocular characteristics compared with the non-ocular sarcoidosis group.

**Table 1:** Demographics and ocular characteristics

	**Control** **(mean ± SD or N [%])**	**Non-ocular sarcoidosis** **(mean ± SD or N [%])**	***p***
**Number of eyes**	30 eyes of 30 patients	30 eyes of 30 patients	
**Demographics**			
Age, years	56.00 ± 11.66	55.13 ± 11.58	0.874
Sex, Male	11 (36.66)	11 (36.66)	1.000
Female	19 (63.33)	19 (63.33)	
Subtypes of sarcoidosis		Pulmonary (27 [90]), Extrapulmonary; skin (3 [10])	
Duration of sarcoidosis, months		16.10 ± 26.18	
Treatment regimen		Prednisolone (6 [20]), MTX (5 [16.66]), Observation (19 [63.63])	
ACE, U/L		51.55 ± 21.77	
CRP, mg/dL		0.20 ± 0.16	
**Ocular characteristics**			
BCVA, logMAR	0.02 ± 0.04	0.03 ± 0.05	0.152
SE, Diopters	-0.80 ± 1.81	-0.71 ± 1.93	0.837
IOP, mmHg	16.06 ± 1.96	15.73 ± 2.40	0.559

*SD* Standard deviation*, MTX* methotrexate*, ACE* angiotensin converting enzyme*, CRP* C-reactive protein*, BCVA* best corrected visual acuity*, SE* spherical equivalent*, IOP* intraocular pressure*, SLCC* Sattler layer-choriocapillaris complex*, HL* Haller layer

**Fig. 3:**
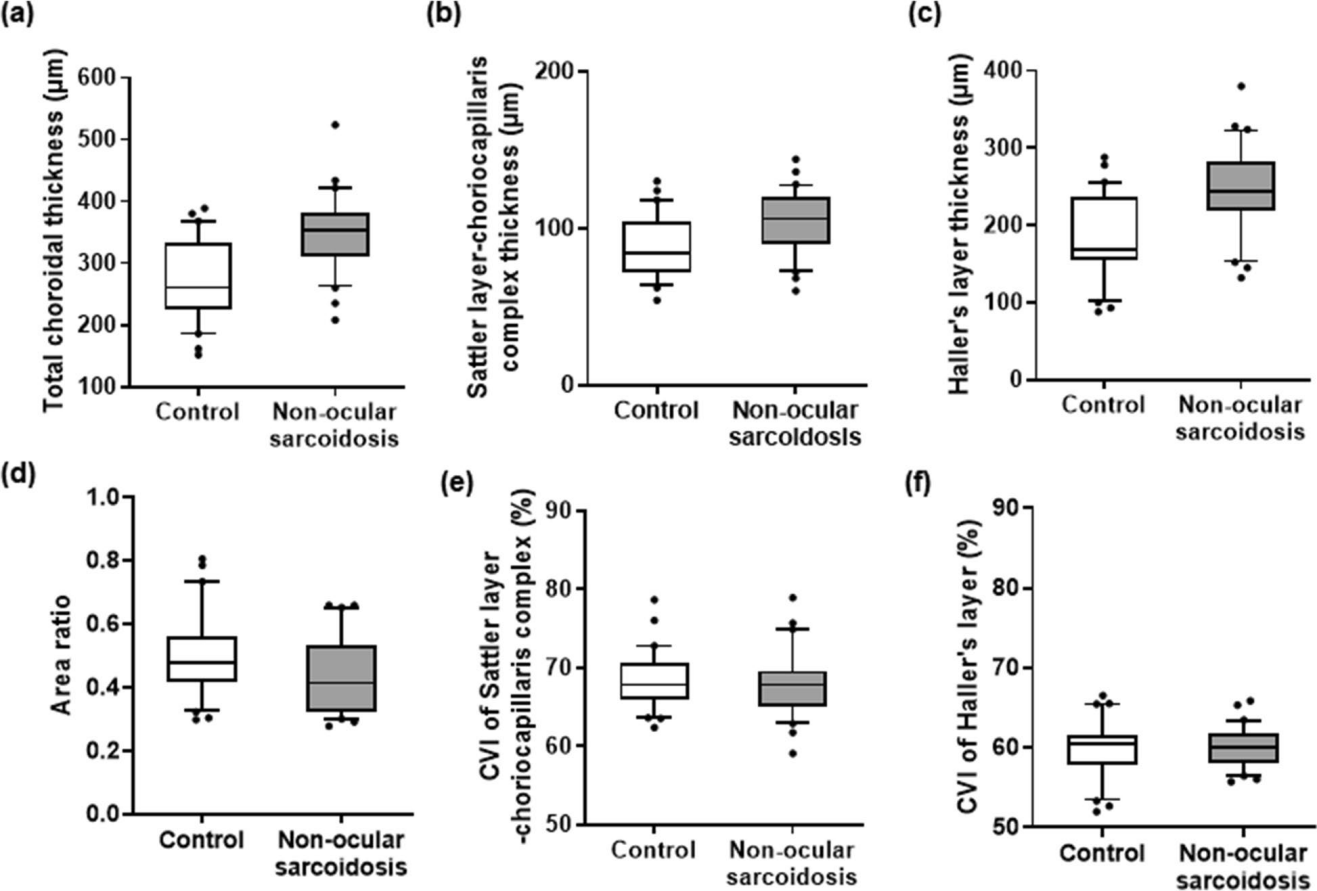
Boxplots of choroidal parameters in healthy control eyes and with non-ocular sarcoidosis eyes. **a** total choroidal thickness (interquartile range [IQR] = 226–332 μm vs. 311–382 μm, median = 260 μm vs. 354 μm) (**b**) Sattler layer-choriocapillaris complex thickness (IQR = 72–104 μm vs. 90–120 μm, median = 84 μm vs. 106 μm), **c** Haller layer thickness (IQR = 154–237 μm vs. 219–283 μm, median = 169 μm vs. 244 μm), **d** choroidal area ratio (IQR = 0.42–0.56 vs. 0.32–0.53, median = 0.47 vs. 0.41), and choroidal vascular index (CVI) of (**e**) Sattler layer-choriocapillaris complex (IQR = 65.97–70.61% vs. 65.03–69.58%, median = 67.82% vs. 67.83%) and (**f**) Haller layer (IQR = 57.80–61.54% vs, 58.16–61.82%, median = 60.48% vs. 60.08%). (box = interquartile range, horizontal line = median, whiskers = 10 to 90 percentile, dots = outliers)

**Fig. 4:**
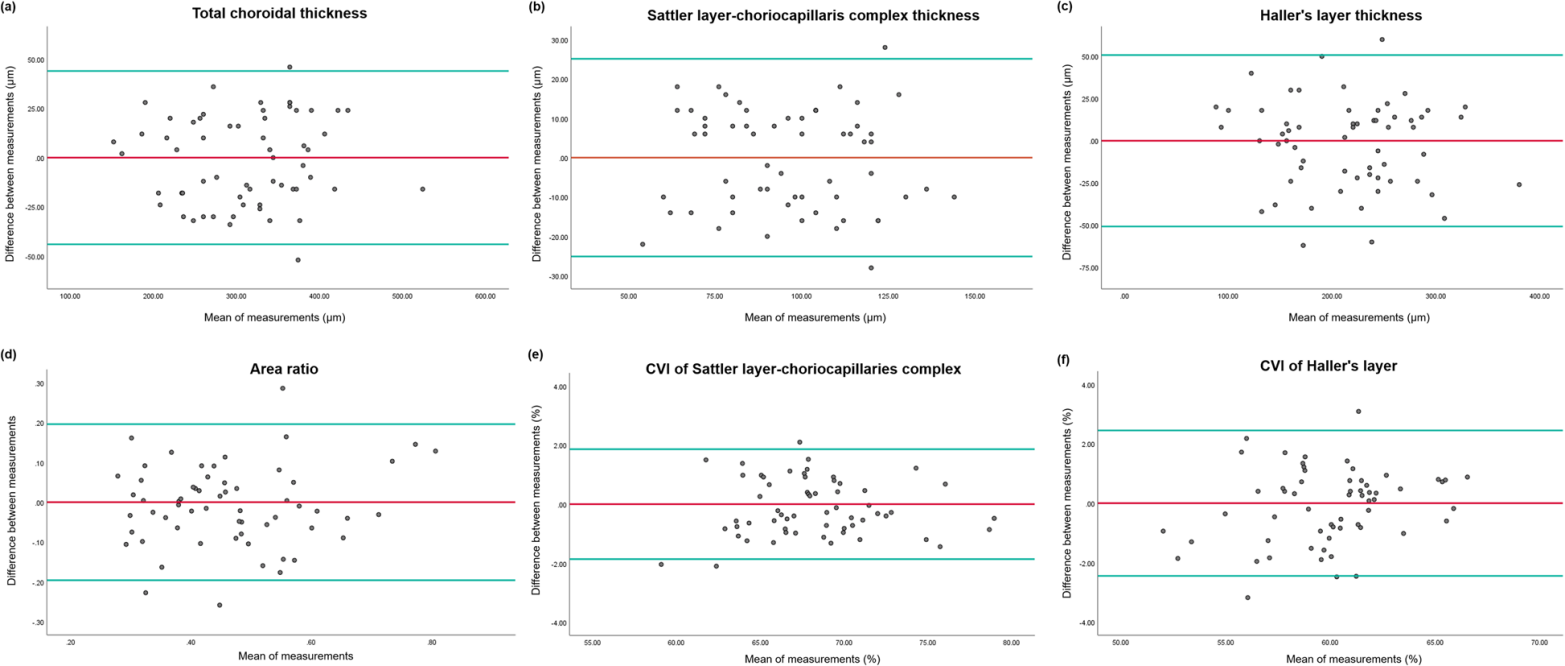
Bland–Altman plots of choroidal measurements in the entire study eyes. **a** total choroidal thickness, **b** Sattler layer-choriocapillaris complex thickness, **c** Haller layer thickness, **d** choroidal area ratio, and choroidal vascular index (CVI) of (**e**) Sattler layer-choriocapillaris complex and (**f**) Haller layer

Choroidal characteristics of are summarized in Table 2. Total and all sublayers (SLCC and HL) were significantly thicker in the non-ocular sarcoidosis group than in the healthy control group (Total: 349.86 ± 62.28 μm vs. 272.13 ± 65.27 μm, *p* < 0.001; SLCC: 103.66 ± 20.28 μm vs. 88.73 ± 20.10 μm, *p* = 0.006; HL: 246.20 ± 54.85 μm vs. 183.40 ± 54.40 μm, *p* < 0.001). The area ratio was significantly lower in the non-ocular sarcoidosis group than the control group (0.43 ± 0.11 vs. 0.50 ± 0.13, *p* = 0.048), indicating the degree of choroidal thickening at HL was greater than SLCC in non-ocular sarcoidosis. There were no significant differences in CVIs of all sublayers between the two groups (SLCC: 67.97 ± 4.30% vs. 68.48 ± 3.67%, *p* = 0.626; HL: 60.03 ± 2.56% vs. 59.89 ± 3.64%, *p* = 0.862). Their distributions are displayed in Fig. 3. The inter-examiner agreements were demonstrated to be good to excellent [17] in all measurements, as assessed by ICC (thickness of total choroid = 0.977 [95% confidence interval, CI = 0.692–0.986, *p* < 0.001], thickness of SLCC = 0.910 [95% CI = 0.850–0.946, *p* < 0.001], thickness of HL = 0.957 [95% CI = 0.929–0.975, *p* < 0.001], the area ratio = 0.839 [95% CI = 0.730–0.904, *p* < 0.001], CVI of SLCC = 0.980 [95% CI = 0.967–0.988, *p* < 0.001], and CVI of HL = 0.960 [95% CI = 0.932–0.976, *p* < 0.001]). The differences between measurements from two examiners were shown by Bland–Altman plots (Fig. 4).

**Table 2:** Choroidal characteristics

	**Control (mean ± SD)**	**Non-ocular sarcoidosis (mean ± SD)**	***p***
**Thickness (μm)**			
Total	272.13 ± 65.27	349.86 ± 62.28	< 0.001**
SLCC	88.73 ± 20.10	103.66 ± 20.28	0.006**
HL	183.40 ± 54.40	246.20 ± 54.85	< 0.001**
**Area ratio**			
SLCC-to-HL	0.50 ± 0.13	0.43 ± 0.11	0.048**
**Choroidal vascularity index (%)**			
SLCC	68.48 ± 3.67	67.97 ± 4.30	0.626
HL	59.89 ± 3.64	60.03 ± 2.56	0.862

*SD* Standard deviation, *SLCC* Sattler layer-choriocapillaris complex, *HL* Haller layer

^**^Statistically significant (*p* < 0.05; Student’s-t test)

In the healthy control group, older age showed a significant association with thinner total choroid (*r*^2^ = 0.200, *p* = 0.013), SLCC (*r*^2^ = 0.183, *p* = 0.018), and HL (*r*^2^ = 0.143, *p* = 0.039) in both univariate and multivariable analysis, whereas, more myopic SE showed its significance only in univariate analysis (total [*r*^2^ = 0.186, *p* = 0.017], SLCC [*r*^2^ = 0.178, *p* = 0.020], and HL [*r*^2^ = 0.130, *p* = 0.049]) (Table 3). In the non-ocular sarcoidosis group, eyes treated with oral steroids showed significantly thinner choroidal thickness than eyes under observation (total choroid: 305.83 μm vs. 360.87 μm, *p* = 0.015; SLCC: 90.33 μm vs. 107.00 μm, *p* = 0.073; and HL: 215.50 μm vs. 253.87 μm, *p* = 0.082) (Table 3). No other systemic or ocular factors were associated with choroidal thickness in both groups.

**Table 3:** Factors associated with choroidal thickness

	**Univariate**						**Multivariable**					
**Control**	**Total**		**SLCC**		**HL**		**Total**		**SLCC**		**HL**	
	***r***^**2**^	***p***	***r***^**2**^	***p***	***r***^**2**^	***p***	***r***^**2**^	***p***	***r***^**2**^	***p***	***r***^**2**^	***p***
**Systemic factors**												
Age	0.200	0.013**	0.183	0.018**	0.143	0.039**	0.200	0.013**	0.183	0.018**	0.143	0.039**
Sex		0.413		0.164		0.880						
**Ocular factors**												
BCVA	0.015	0.520	0.034	0.327	0.006	0.682		0.775		0.503		0.928
SE	0.186	0.017**	0.178	0.020**	0.130	0.049**		0.246		0.242		0.358
IOP	0.067	0.167	0.066	0.172	0.047	0.252		0.197		0.308		0.415
**Non-ocular sarcoidosis**	**Total**		**SLCC**		**HL**		**Total**		**SLCC**		**HL**	
	***r***^**2**^	***p***	***r***^**2**^	***p***	***r***^**2**^	***p***	***r***^**2**^	***p***	***r***^**2**^	***p***	***r***^**2**^	***p***
**Systemic factors**												
Age	0.001	0.870	0.000	0.990	0.001	0.857	0.175	0.636	0.154	0.893	0.191	0.549
Sex		0.363		0.194		0.579						
Subtypes of sarcoidosis												
Pulmonary vs. Extra-pulmonary		0.912		0.748		0.870						
Duration of sarcoidosis	0.052	0.224	0.005	0.724	0.055	0.211		0.516		0.987		0.450
Treatment regimen												
Steroid vs. observation		0.015**		0.073*		0.082*						
Methotrexate vs. observation		0.327		0.787		0.327						
ACE	0.076	0.149	0.020	0.461	0.072	0.161		0.405		0.869		0.366
CRP	0.022	0.441	0.056	0.215	0.007	0.676		0.726		0.105		0.898
**Ocular factors**												
BCVA	0.002	0.819	0.030	0.364	0.013	0.552		0.727		0.412		0.914
SE	0.035	0.323	0.014	0.535	0.028	0.373		0.209		0.965		0.150
IOP	0.017	0.495	0.003	0.780	0.016	0.502		0.874		0.478		0.941

*SLCC* Sattler layer-choriocapillaris complex, *HL* Haller layer, *ACE* angiotensin converting enzyme, *CRP* C-reactive protein, *BCVA* best corrected visual acuity*, SE* spherical equivalent, *IOP* intraocular pressure

^**^Statistically significant (*p* < 0.05) and *borderline significant (*p* < 0.1); Linear regression analysis for continuous variable and Chi-squared test for categorical variables

## Discussion

In this study, all sublayers of the subfoveal choroid were significantly thicker, especially HL, without vascularity changes in non-ocular sarcoidosis eyes compared with healthy control eyes. To the best of our knowledge, this is the first study to demonstrate subclinical structural alterations of the choroid using EDI SD-OCT in eyes with systemic sarcoidosis without overt ocular involvement.

With the advancement of OCT technology allowing in-vivo cross-sectional visualization of the entire choroid, changes in choroidal thickness in ocular sarcoidosis have been reported. Mehta et al. [7] demonstrated a disproportionately enlarged Sattler layer in sarcoidosis-related uveitis and choroidal thickening in active granulomatous uveitis. On the other hand, Güngör et al. [6] reported that patients with ocular sarcoidosis had a thinner subfoveal choroid during the quiescent phases than normal controls. The choroid is supposed to be highly reactive to inflammatory conditions due to its highly vascularized structure without the autoregulation of perfusion [18]. Pro-inflammatory cytokines, such as prostaglandins, are known to be upregulated and have central roles in systemic sarcoidosis [19–21]. It is speculated that inflammatory reaction of the choroidal vascular network to pro-inflammatory cytokines is the main mechanism of increasing choroidal thickness in intraocular inflammation associated with sarcoidosis [6]. However chronic choroidal inflammation ultimately leads to choroidal atrophy and thinning in the quiescent phase [7].

The thicknesses and CVIs of the total and each sublayer of the choroid were within the normal range in both groups of this study, based on results from large cohort studies [15, 22]. However, there were notable relative differences in choroidal thickness between non-ocular sarcoidosis and healthy control groups. Specifically, total and all sublayers of the choroid were thicker in non-ocular sarcoidosis group compared to healthy control group. Moreover, the degree of choroidal thickening in non-ocular sarcoidosis group was significantly greater at HL than at SLCC, which contrasts with a previous report indicating predominant Sattler layer thickening in active sarcoid-related uveitis [7]. Furthermore, although it is reported that CVI increases along with choroidal enlargement in active intraocular inflammation, such as human leukocyte antigen-B26 related uveitis [23], no significant changes in CVIs were observed across all sublayers in non-ocular sarcoidosis group compared with the control group in this study. These findings suggest that choroidal thickening in all sublayers, especially HL, without changes in vascularity, is a characteristic choroidal manifestation of non-ocular sarcoidosis. We suspect that non-ocular sarcoidosis may be under subclinical inflammation, without apparent signs of intraocular inflammation, resulting in both vasodilation and stromal edema (increase of both luminal and stromal area), thereby leading to choroidal expansion without changes in vascular density. These characteristic choroidal alterations of non-ocular sarcoidosis captured on EDI SD-OCT may shed light on the pathophysiological understanding of how sarcoidosis initiates sarcoidosis-associated ocular inflammation from subclinical stage. It also implies that eyes may be more often affected by systemic sarcoidosis than previously known, considering subclinical changes.

Several reports demonstrated that a subsequent decrease in choroid thickness after steroid treatments when it is initially increased in response to the intraocular inflammation in conditions such as human leukocyte antigen-B27 related uveitis, Behçet's disease, and Vogt-Koyanagi-Harada disease [24–26]. Notably, in this non-ocular sarcoidosis group, steroid treatment also showed a significant association with choroidal thickness; Eyes treated with oral steroids had thinner choroid compared with eyes under observation. This choroidal response to the steroid treatment may further support that eyes with non-ocular sarcoidosis are under subclinical choroidal inflammation. Unfortunately, we could not identify serum biomarkers reflecting localized and subclinical choroidal inflammation status in eyes with non-ocular sarcoidosis; Choroidal thickness in the non-ocular sarcoidosis group did not show significant association with CRP (an inflammation marker in various inflammatory systemic diseases) [27–29] or ACE (a biomarker for systemic sarcoidosis, correlated with the granuloma burden and disease severity) levels [30, 31]. Caution is needed in interpreting these results due to a time gap between blood tests and OCT acquisition caused by referral-to-ophthalmologic screening duration (mean 33.38 ± 41.12 days). Further prospective studies, including additional blood test components besides CRP and ACE, are necessary for validation of this issue.

Age and axial length are considered as significant factors influencing choroidal thickness, with older age and longer axial length associated with reduced total choroidal thickness [22]. However, in this study, a significant association between age and choroidal thickness was found only in healthy control eyes, not in those with non-ocular sarcoidosis. Furthermore, SE, which is expected to reflect axial length, also showed a significant association with choroidal thickness only in healthy control, not in non-ocular sarcoidosis. These findings suggest that pro-inflammatory environment of systemic sarcoidosis may exert a more substantial influence on choroidal thickness than age or axial length alone. While some studies [32, 33] have reported a decrease in choroidal thickness proportional to the rise in intraocular pressure, and others [34, 35] have suggested no correlation, this study found no significant association between intraocular pressure and choroidal thickness in both groups.

Our study had several limitations. First is a small sample size. Second is the retrospective study design. Thus, it was impossible to adjust all known confounding factors between groups, most importantly, axial length and diurnal variation. SE-matching and OCT image acquisition time-matching within 2 h difference may help mitigate this issue. Third is manual segmentation and measurement. The participation of two independent examiners and binarization at the standard threshold might overcome these challenges. Nonetheless, this study is meaningful because this is the first investigation of choroidal changes associated with systemic sarcoidosis without overt ocular sarcoidosis using EDI SD-OCT. The more detailed morphological characteristics of the choroidal vasculature in non-ocular sarcoidosis can be explored in further studies using wICGA analysis. A large-scale prospective longitudinal study is required to clarify whether the subclinical choroidal thickening in non-ocular sarcoidosis is a pre-inflammatory sign by evaluating whether they progress to overt ocular inflammation.

In conclusion, this study demonstrated that systemic sarcoidosis may have subclinical manifestations in the choroid, resulting in total and all sublayers thickening, especially HL, without significant vascularity changes. Early ophthalmologic screening using EDI-SD OCT is important to detect these subclinical choroidal changes in systemic sarcoidosis patients.

## Data Availability

The datasets used and/or analyzed during the current study are available from the corresponding author on reasonable request.
